# Quality of life and severity of symptoms among patients with various degrees of reflux esophagitis: a prospective study

**DOI:** 10.1038/s41598-023-41332-w

**Published:** 2023-08-26

**Authors:** Amir Mari, Wasef Na’amnih, Loay Ghantous, Helal Said Ahmad, Tawfik Khoury, Khitam Muhsen

**Affiliations:** 1 Gastroenterology Department, Azrieli Faculty of Medicine, Gastroenterology and Hepatology Institute, Nazareth Hospital, Bar Ilan University, 16100 Nazareth, Israel; 2 Department of Epidemiology and Preventive Medicine, School of Public Health, Faculty of Medicine, Tel Aviv University, Tel Aviv, Israel; 3 Internal Medicine Department, Azrieli Faculty of Medicine, Nazareth Hospital, Bar Ilan University, Nazareth, Israel

**Keywords:** Oesophagogastroscopy, Gastroenterology, Health care

## Abstract

Gastro-esophageal reflux disease (GERD) can cause erosive esophagitis (EE) and compromise the quality of life (QoL). We examined differences in symptom severity and QoL according to EE severity grade. A follow-up study was conducted among GERD patients at the Nazareth Hospital in Israel. Patients underwent a baseline gastroscopy in 2014–2020 during which the EE grade was determined using the Los Angeles classification. Follow-up telephone interviews were conducted during 2019–2020 with a mean time interval of 18.9 months (SD = 14.9) after the baseline gastroscopy to assess GERD symptoms using the Reflux disease questionnaire (RDQ) and QoL using the GERD QoL questionnaire. The patients were interviewed in their native language (Arabic or Hebrew). Overall, 149 (66.4% males) patients were included; 50 had EE grades C/D and 99 had grades A/B. The mean age at baseline and follow-up was 44.6 years (SD = 15.1) and 46.2 years (SD = 14.9), respectively. Cronbach’s alpha was 0.928 and 0.855 for the RDQ and QoL questionnaires, respectively. Patients with EE C/D grades had more severe symptoms than patients with EE A/B grades (*P* = 0.05), especially in regurgitation scores (*P* = 0.03). Females had more severe symptoms (overall) than males (adjusted OR = 2.34; 95% CI 1.12–4.90). Patients with the more severe esophagitis EE C/D group (adjusted OR = 1.98; 95% CI 0.93–4.24) and those who used PPIs treatment (adjusted OR = 2.19; 95% CI 0.95–5.01) reported more severe GERD symptoms. The number of schooling years was significantly associated with better QoL score (beta coefficient 1.33, *P* = 0.005) but not EE grade or GERD symptoms. Follow-up endoscopy conducted among 22 patients with EE grades C/D showed that 13 (59.1%) of these patients had normal endoscopic findings, 6 patients (27.3%) had a grade A EE, 1 patient (4.5%) had grade B, and 2 (9.1%) remained with grade C EE. The Arabic and Hebrew versions of the RDQ and QoL questionnaires were highly reliable. GERD symptoms severity was more profound among patients with more severe esophagitis. No significant association between EE grade and QoL; this negative result might be due to the improvement in esophagitis endoscopic findings among patients with C/D grade.

## Introduction

Gastroesophageal reflux disease (GERD) is defined as symptoms or complications resulting from the reflux of gastric contents into the esophagus or beyond, into the oral cavity (including the larynx) or lung^1^. GERD is the most frequent gastrointestinal-related diagnosis made in the United States^2^. The estimated prevalence of GERD in Western countries is between 18.1 and 27.8%^3^. The core symptoms of GERD are heartburn and regurgitation and non-cardiac chest pain^4,5^. The Los Angeles scale is the most commonly used classification system for grading reflux esophagitis severity^6,7^, which classifies esophagitis stages from A to D, when D is the most severe disease^6^. Medical therapy with proton pump inhibitors (PPIs) is considered the most effective therapy for GERD, due to their profound and consistent acid suppression^8^.

GERD symptoms might negatively impact patients' QoL in terms of physical, social, and emotional well-being^9,10^. The negative effect of GERD on QOL is becoming better recognized given the use of patient-reported outcome instruments to study the influence of GERD symptoms^10^. The German Pro GERD study showed that GERD symptoms impair patients’ QoL, both physical and psychosocial aspects of well-being compared with the general population^11^. A study conducted in Sweden assessed the impact of GERD symptoms on QoL and found that even patients with mild GERD symptoms had reduced well-being and QoL^12^. A large study of 12,815 GERD patients from six European countries showed that GERD symptoms were associated with a substantial impact on the daily living activities^13^. Other studies from the Middle East, from Iran^14^ and Saudi Arabia^15^, also reported a reduced QoL in GERD patients. Nonetheless it is unclear whether the impact of GERD on QoL is related to the esophagitis degree or symptoms severity. Accordingly, we assessed symptoms severity and quality of life in patients with severe reflux esophagitis (grades C/D) compared to patients with mild/moderate reflux esophagitis (grades A/B). Our hypothesis was that the severity degree of esophagitis might be related to QoL in GERD patients.

## Methods

### Study design and population

This study was conducted in the Nazareth hospital that serves the population in the Nazareth area, in the north of Israel. The population in this region includes Jewish and Arab residents, commonly living in distinct communities. According to the Israel Central Bureau of Statistics in 2020, the population in the north region 1,479,800 residents, of whom 794,900 (53.7%) were Arabs (mostly Muslims, 72.9%), 5629,400 (42.5%) were Jews, and 3.8% belonged to other ethnicities^16^. All Israeli citizens have universal healthcare insurance according to the National Health Insurance Law implemented since 1995^17^. Primary care is generally available in all towns and villages with high accessibility to all and is served by four health maintenance organizations. Treatment in hospitals and large specialists’ clinics as well as medications and diagnostic tests are all covered by the universal healthcare insurance. Typically, referrals to hospitals and specialized clinics are done by primary care physicians in return for a financial commitment by the health maintenance organization.

This follow-up study was undertaken among patients with GERD at the gastroenterology department Nazareth Hospital, a-150 beds regional teaching hospital at Nazareth city.

GERD patients who underwent a baseline endoscopy during 2014–2020 and classified as having EE grades A-D using the Los Angeles classification, were contacted by phone and invited to participate in the follow-up study during 2019–2020 as previously described^18,19^. Individuals who provided informed consent by phone were interviewed to collect information on demographics, symptoms severity and QoL using structured questionnaires. We approached all patients with EE grades C/D and a randomly selected sample of patients of EE grades A/B^19^. The mean time between the endoscopy and the follow up call was 18.9 months (SD = 14.9).

### Definition of the study variables

The dependent variables were

1. QoL—it was assessed using the GERD QoL questionnaire, a 16– item questionnaire, designed to assess the impact of GERD symptoms on sleep, exercise, work, social activities, diet, treatment effect, sex life, and psychological well-being^20^. The patients were asked to rate their agreement with each item on a Likert scale as follows: strongly agree, somewhat agree, neutral, somewhat disagree, and strongly disagree.
2. Symptoms severity: GERD symptoms severity was assessed using the reflux disease questionnaire (RDQ), a 12-item questionnaire, designed to assess the frequency and severity of heartburn, regurgitation, and dyspeptic complaints during the last week prior to the interview^21^. The questionnaire is based on a Likert scale with responses/scores ranging from 0 to 5 for frequency (not present to present daily) and severity (not present to severe). Using the participant’s replies his/her score was calculated for each subclass of symptoms (heartburn, regurgitation, and dyspepsia) as the mean of item responses^21^, with higher scores indicating more severe or frequent symptoms.

The RDQ and QoL questionnaire was translated to Arabic and Hebrew by the study researchers who speak both languages at native level. The face and content validity of the questionnaires were assessed. The clarity of the translated questions was tested and discussed by experts until consensus reached. All translation and back translation were achieved prior to study initiation. A pilot study of 10 patients was completed before administering the study questionnaire for all participants to assess feasibility and acceptance.

#### The independent variable

The main independent variable was the severity degree of EE: as defined at baseline gastroscopy using the Los Angeles grading system: grade C/D vs. grade A/B: a dichotomous variable.

*Co-variates*. Socio-demographic factors (age at baseline in years, sex, number of schooling years, population group), lifestyle factors (smoking, alcohol consumption, physical activity for 30 min at least once a week, and body mass index [BMI]—defined based on reported weight and height), comorbidity that defined using the Charlson's comorbidity index^22^, and proton pump inhibitors (PPIs) treatment. All co-variates were defined based on self-reports and the reported medical data were validated against the medical records.

### Statistical analysis

Assessment of the reliability of the GERD QoL questionnaire and the RDQ were performed using Cronbach’s alpha for evaluating the internal consistency of the sub-scales and overall scale.

The participants' demographics and clinical characteristics were described using mean and standard deviations (SD) for continuous variables and counts and percentages for categorical variables. The student’s *t* test was used to assess the associations of EE grades with quality of life (based on the questionnaire score and the Mann–Whitney *U* test was used for variables with skewed distribution. The chi-square or Fisher exact test were used for categorical variables. Odds ratios (OR) and 95% confidence intervals (CI) were calculated for the independent variables, from bivariate and multivariable logistic regression models, in which overall symptoms severity score (above median vs. median and below score) was the dependent variable. The correlations between QoL score and various covariates were examined using the Pearson and Spearman’s correlation coefficients, separately for patients with or without symptoms of GERD. Multivariable linear regression models that adjusted for symptoms of GERD were used to assess associations of age at baseline (years), sex, schooling years, esophagitis severity, PPIs treatment, physical activity for 30 min once a week, and overall GERD symptoms severity scale and QoL score. All statistical tests were two-sided, and *P* < 0.05 was considered statistically significant. Data analysis was performed using Statistical Package for the Social Science (SPSS) version 27 (IBM, Armonk, New York, USA).

### Ethical aspects

The study protocol was approved by the Institutional Review Board (IRB) at the Nazareth hospital. The study was performed in accordance with relevant guidelines and regulations. The participants were given a detailed explanation by the student on the study in their native language (Arabic or Hebrew) and were be asked by phone to provide an informed consent.

## Results

Overall GERD 224 patients were successfully contacted and asked to participate in the study, of those 149 patients agreed to participate (response rate 65.5%). The mean age of these 149 patients, (66.4% males) was 44.6 years (SD = 15.1) and 46.2 years (SD = 14.9) at the baseline and the follow-up endoscopies, respectively. Most participants (92.6%) were Arab patients and 7.4% were Jews. Overall 99 (66.4%) of the participants had mild-moderate EE (grades A/B) and 50 (33.6%) had severe EE (grades C/D). There were no significant differences between participants with EE grades A/B and those with grades C/D in the mean age at baseline, population group, BMI, smoking, alcohol consumption, physical activity and Charlson's index. The proportion of males was slightly higher in the C/D group, compared to participants with EE grades A/B (*P* = 0.08). Participants with EE grades C/D had a lower mean number of schooling years vs. participants with EE grades A/B (*P* = 0.03) (Table 1). Overall 61 (61.6%) participants with EE-A/B were treated with PPI vs. 43 (86.0%) patients with EE-C/D (*P* = 0.002) (Table 1). The mean duration of PPI treatment among patients with EE grades C/D was 3.4 months (SD = 8.7; range 1–12 months).Prescription of additional drugs was documented in 6 patients who received H2 blockers (Famotidine). Moreover, 22 (44%) of the patients with EE grades C/D underwent a follow up endoscopy. Results of the follow-up endoscopy conducted among 22 patients with EE grades C/D showed that most patients improved; 13 (59.1%) of these patients had normal endoscopic findings, 6 patients (27.3%) had a grade A esophagitis, 1 patient (4.5%) had grade B esophagitis, while 2 patients (9.1%) remained with grade C esophagitis.

**Table 1:** Characteristics of study participants by severity of reflux esophagitis.

	EE grades A/B (N = 99)	EE grades C/D (N = 50)	*P* value*
Mean age at baseline (years), (SD)	43.7 (14.3)	46.0 (16.3)	0.4
Number of schooling years mean, (SD)	12.1 (3.3)	10.8 (3.2)	0.03
Charlson’s index, mean, (SD)	1.32 (2.1)	2.08 (3.1)	0.1
Sex			0.08
Male	61 (61.6%)	38 (76.0%)	
Female	38 (38.4%)	12 (24.0%)	
Population group			0.4
Arabs	93 (93.9%)	45 (90.0%)	
Jews	6 (6.1%)	5 (10.0%)	
BMI (categorical)kg/m^2^			0.1
BMI 20–24 (normal)	24 (24.5%)	6 (12.0%)	
BMI 25–29 (overweight)	48 (49.0%)	24 (48.0%)	
BMI ≥ 30 (obesity)	26 (26.5%)	20 (40.0%)	
Smoking			0.3
Yes (past and current)	37 (37.4%)	23 (46.0%)	
No	62 (62.6%)	27 (54.0%)	
Alcohol consumption			0.6
Yes	9 (9.1%)	6 (12.0%)	
No	90 (90.9%)	44 (88.0%)	
Physical activity for 30 min at least once a week			0.9
Yes	31 (31.3%)	16 (32.0%)	
No	68 (68.7%)	34 (68.0%)	
PPIs treatment			0.002
Yes	61 (61.6%)	43 (86.0%)	
No	38 (38.4%)	7 (14.0%)	

*BMI* body mass index, *PPIs* Proton pump inhibitors, *SD* standard deviation, *EE* erosive esophagitis, *kg* kilogram, *m* meters.

*P value was obtained by the chi-square test for categorical variables and Mann–Whitney for continuous variables.

### Validity of the RDQ and QoL questionnaires

The study investigators rated the face and content validity of the translated questionnaires as good. Overall 138 patients filled in the Arabic version and 11 patients filled in the Hebrew version questionnaires. The reliability of the translated RDQ was excellent with Cronbach’s alpha of 0.928 for the 12-item questionnaire. Split of the RDQ into two halves showed good reliability with Cronbach’s alpha of 0.858 (for items 1–6) and for items 7–12 (Cronbach’s alpha 0.844). The Cronbach’s alpha of the translated GERD QoL questionnaire was 0.855. The Cronbach’s alpha for each domain was as follow: diet and food intake = 0.751, daily activity = 0.762, medications = 0.674, and psychosocial aspects = 0.758.

### Severity of symptoms according to EE grades

The overall symptoms severity scores slightly differed between the two esophagitis groups (*P* = 0.05). More patients with esophagitis grades C/D had a score of above the median in the regurgitation (54.0 vs. 35.4%, *P* = 0.03) and heartburn (56.0 vs. 40.4%, *P* = 0.07) scales compared to patients with EE-A/B. The score of the dyspepsia did not differ significantly between the two esophagitis groups (*P* = 0.2) (Table 2).

**Table 2:** Comparison of severity scores of symptoms using the RDQ between patients with EE grades C/D and those with grade A/B.

	EE grades A/B (N = 99)	EE grades C/D (N = 50)	*P* value^a^
Overall symptoms severity scale			0.04
Above median	38 (38.4%)	28 (56.0%)	
Median and below	61 (61.6%)	22 (44.0%)	
Regurgitation scale			0.03
Above median	35 (35.4%)	27 (54.0%)	
Median and below	64 (64.6%)	23 (46.0%)	
Heartburn scale			0.07
Above median	40 (40.4%)	28 (56.0%)	
Median and below	59 (59.6%)	22 (44.0%)	
Dyspepsia scale			0.2
Above median	37 (37.4%)	24 (48.0%)	
Median and below	62 (62.6%)	26 (52.0%)	

*EE* erosive esophagitis.

^a^*P* value by the chi square test where appropriate for categorical variable.

We re-analyzed these data using a different approach. Namely we assessed differences between patients with EE grades A/B vs. grades C/D in the median score of overall symptoms severity and the scores of symptoms sub-scales. This analysis showed supportive findings of significant difference between the groups mainly in regurgitation score (supplementary Table 1).

Bivariate analysis and multivariable logistic regression models were performed to assess the association between overall GERD symptoms severity and other variables including sex (female vs. male), esophagitis severity group (A/B vs. C/D), PPIs treatment (yes vs. no), age at baseline (years), the Charlson’s comorbidity index and the number of schooling years. Females compared to males had more severe overall symptoms severity (adjusted OR = 2.34; 95% CI 1.12–4.90, *P* = 0.02). Moreover, patients with the more severe esophagitis EE C/D group (adjusted OR = 1.98; 95% CI 0.93–4.24, *P* = 0.08) and who use PPIs treatment (adjusted OR = 2.19; 95% CI 0.95–5.01, *P* = 0.07) reported more severe GERD symptoms. The other variables in the multivariable model were not associated with symptoms severity; age (*P* = 0.08), Charlson’s co-morbidity index (*P* = 0.7), and schooling years (*P* = 0.4) (Table 3).

**Table 3:** Logistic regression model of factors associated with overall GERD symptoms severity.

	Unadjusted OR (95% CI)	*P* value^a^	Adjusted OR (95% CI)	*P* value^b^
Age at baseline (years), a continuous variable	0.99 (0.97–1.01)	0.4	0.97 (0.94–1.00)	0.08
Sex: female vs. male	2.04 (1.03–4.07)	0.04	2.34 (1.12–4.90)	0.02
Number of schooling years, a continuous variable	0.96 (0.87–1.06)	0.4	0.95 (0.84–1.07)	0.4
Esophagitis severity; (C/D vs. A/B)	2.04 (1.02–4.07)	0.04	1.98 (0.93–4.24)	0.08
Chalrson’s comorbidity index, a continuous variable	1.01 (0.89–1.15)	0.8	1.04 (0.87–1.24)	0.7
PPIs treatment (Yes vs. no)	2.21 (1.06–4.64)	0.03	2.19 (0.95–5.01)	0.07

*CI* confidence interval, *OR* odds ratio, *PPIs* proton pump inhibitors.

^a^*P* value was obtained from bivariate logistic regression model.

^b^*P* value was obtained from multiple logistic regression model.

### Quality of life

The overall mean QoL score was 24.56 (SD = 19.04). No significant differences were found in the overall QoL score or in QoL subclasses (daily activity, treatment effects, diet effect or psychological wellbeing) according to esophagitis grades (Table 4).

**Table 4:** Quality of life among patients with EE grades C/D and those with grades A/B.

	EE grades A/B (N = 99)	EE grades C/D (N = 50)	*P* value^a^
Overall QoL score (mean) (SD)	23.0 (16.0)	28.1 (18.9)	0.6
QoL scores for subclasses			
Daily activity	26.0 (15.1)	28.1 (18.9)	0.2
Treatment effects	11.4 (18.6)	15.0 (21.6)	0.3
Dietary effects	39.4 (31.0)	42.1 (32.8)	0.9
Psychosocial wellbeing	17.2 (26.5)	20.7 (29.1)	0.7

*SD*, standard deviation, *QoL* quality of life.

^a^*P* value by the Mann–Whitney for continuous/discrete variables.

Among patients (N = 54) who reported GERD symptoms in the week before the follow-up interview, the overall QoL score was weakly correlated with schooling years, but this correlation was not statistically significant (r = 0.178, *P* = 0.08). Among patients who did not report GERD symptoms (N = 95) in week before the interview, a significant moderate positive correlation was found between overall QoL score and schooling years (r = 0.329, *P* = 0.02). Age at baseline, sex, esophagitis severity, PPIs treatment, physical activity and overall GERD symptoms severity were not correlated with overall QoL score (Table 5).

**Table 5:** Correlations of demographic and clinical factors with Quality of Life by symptoms of GERD.

	Overall QoL			
	With GERD symptoms# N = 54		Without GERD symptoms# N = 95	
	Correlation coefficient	*P* value	Correlation coefficient	*P* value
Age at baseline (years), a continuous variable	− 0.049^a^	0.6	− 0.142^a^	0.3
Sex (female vs. male)	− 0.116	0.3	0.128	0.4
Schooling years, a continuous variable	0.178^a^	0.08	0.329^a^	0.02
Esophagitis severity (C/D vs. A/B)	0.039	0.7	− 0.073	0.6
PPIs treatment (Yes vs. no)	− 0.031	0.7	0.027	0.8
Physical activity for 30 min once a week (Yes vs. no)	0.090	0.4	0.099	0.5
Overall GERD symptoms severity scale (Above median vs. median and below)	0.166	0.1	NS	NS

*PPIs* proton pump inhibitors, *QoL* quality of life.

^a^Pearson correlation coefficient.

#Symptoms in the week before the follow-up interview. Data presented are Spearman’s correlation coefficient unless specified otherwise.

A multivariable linear regression model showed no significant association between esophagitis severity grade and overall QoL score (beta coefficient 2.58, *P* = 0.4), sex (beta coefficient − 3.82, *P* = 0.2) and overall GERD symptoms severity scale (beta coefficient 5.23, *P* = 0.1). The number of schooling years (beta coefficient 1.33, *P* = 0.005) was significantly associated with better QoL score (Table 6).

**Table 6:** Multivariable linear regression model of factors associated with overall QoL.

	Overall QoL	
	Unstandardized Beta (95%CI)	*P* value
Age at baseline (years), a continuous variable	0.06 (− 0.15–0.26)	0.6
Sex (female vs. male)	− 3.82 (− 9.54–1.90)	0.2
Schooling years, a continuous variable	1.33 (0.41–2.24)	0.005
Esophagitis severity (C/D vs. A/B)	2.58 (− 3.28–8.44)	0.4
PPIs treatment (Yes vs. no)	− 0.23 (− 6.46–6.00)	0.9
Physical activity for 30 min once a week (Yes vs. no)	3.47 (− 2.31–9.25)	0.2
Overall GERD symptoms severity scale (Above median vs. median and below)	5.23 (− 1.90–12.36)	0.1

*CI* confidence interval, *PPIs* proton pump inhibitors, *QoL* quality of life.

Adjusted R square for model 0.21. The model was adjusted by symptoms of GERD (Yes vs. no).

## Discussion

We showed good validity and reliability of the Arabic and Hebrew versions of the RDQ and QoL questionnaire and the translated versions can be implemented in clinical practice in Israeli and Arab populations. Our results are in agreement with previous studies in which the RDQ was translated to several languages with good validity and reliability, such as Italian^23^, Spanish^24^, Chinese^25^ and Turkish^26^. We have shown acceptable validity and reliability of the Arabic and Hebrew versions of the QoL questionnaire. In agreement with our results, the QoL questionnaire was translated from Chinese language so far, only to English language, with good validity and reliability^20^. To our knowledge, this is the first study that translated and validated the QoL questionnaire after its development in Chinese language and translation to English language. We believe that the clinical application of these questionnaires is mainly for clinical follow up and the evaluation of treatment response.

We found more severe overall GERD symptoms and especially regurgitation among patients with the more severe esophagitis i.e., EE C/D grade. This could be explained by the fact that regurgitation is a classical prototypical symptom of true GERD, more than dyspepsia and heartburn that may represent other conditions like functional heartburn or non-erosive reflux disease^27^. We expected a stronger association between symptoms severity and esophagitis grade. Nonetheless, a possible explanation for this observation is the fact that esophagitis grade C/D is diagnostic for true GERD according to the Lyon consensus, whereas esophagitis grade A/B is a less specific endoscopic finding and is not diagnostic for true GERD^26^. Furthermore, large overlap between esophagitis A/B and functional esophageal conditions have been observed, meaning that the EE-A/B patients could have a substantial functional or hypersensitive component producing a comparable symptoms severity to the more severe GERD patients^28^. Most patients with EE grades C/D who performed a follow-up endoscopy in our study showed healing and down-staging of baseline endoscopic findings, which might explain the weaker than expected correlation that we found between baseline EE grade and symptoms severity at the follow-up as determined by the RDQ. Nason et al. in their study conducted among 769 GERD patients found a positive association between symptoms severity and the presence of erosive esophagitis compared to those with mild or no symptoms, however their study lacked stratification to the different degrees of erosive esophagitis^29^. Previous studies reported inconsistent results regarding the possible correlation between GERD symptoms severity and the presence of GERD mucosal complications such as Barrett’s esophagus. Locke et al.^30^ found no association between Barrett’s esophagus and GERD symptom severity in a large community-based population referred for gastroscopy, whereas Eloubeidi et al.^31^ reported that patients with Barrett’s esophagus were less likely to report worse symptoms than patients with clinical GERD and no Barrett’s. A multi-centre study from Japan^32^ evaluated the correlation between GERD symptoms and endoscopic findings of 8031 subjects, using the endoscopic Los Angeles classification, showed that 40% of the severe esophagitis EE-C/D patients did not complain of any symptoms, although no comparison between the different esophagitis groups was made. Vakil et al.^33^ in their study of 11,945 patients with an endoscopic confirmed erosive esophagitis (Los Angeles grades A–D) reported that 43% of patients with severe esophagitis had dysphagia (probably representing more severe symptoms), compared to 35% of patients with mild esophagitis. Due to the high prevalence of dysphagia in both esophagitis groups, the authors concluded that dysphagia is unreliable predictor of esophagitis severity. To our knowledge no studies have assessed the correlation between GERD symptoms severity using a validated questionnaire and the degree of esophagitis based on the Los Angeles classification, thus our study provides novel findings. A community-based study from Australia, which evaluated the impact of sex in GERD symptoms severity found that female patients had more frequent and severe heartburn and regurgitations compared to male patients^34^. These results are in line with our results where female patients had worse GERD symptoms including heartburn and dyspepsia. This finding may reflect dissimilar awareness and perception of symptoms between genders^34^.

We found no significant association between EE grade and QoL, which mirrors negative results in this component of the study. Thus our hypothesis that more severe esophagitis patients would have poorer QoL was not confirmed. This discrepancy might be explained by the fact that patients with more severe esophagitis did not have significantly more profound severe symptoms, as already discussed. An additional explanation might be that the GERD specific QoL questionnaire might not fully capture all aspects of QoL. Thus, a future research direction following this preliminary validation study needs to include additional general QoL questionnaires such as SF-36 questionnaire. A study from Poland hat included 118 GERD patients assessed the effect of symptoms severity on QoL (using the SF-36 instrument) found that only symptoms frequency was associated with poorer QoL^35^. In our study, only schooling years and sex were independently associated with QoL. Unsurprisingly, higher education level improves the general health related QoL^36^. According to the Saudi Arabian study^15^ and the Polish study^36^, education level was not associated with QoL in GERD patients. Moreover, low level of education was shown to be a risk factor for GERD symptoms appearance as reported in a prospective study of 29,610 GERD patients from Norway^37^. Women had poorer QoL than men, while other studies^34–37^ were inconsistent regarding the association between sex and QoL among GERD patients.

Our study has some limitations. Most study variables were defined based on self-reports, therefore reporting bias on some variables (e.g., income, education and alcohol consumption) might be present; however, we do not assume that reporting bias might be related to EE grade or GERD symptoms. Moreover, our study included patients with EE only, which might represent a special group of GERD, which may affect the generalizability of our findings. Furthermore, the sample size of patients with grades C/D who underwent follow-up endoscopy was small, which did not enable sub-group analysis by follow-up endoscopic improvement status. Patients received clear explanations before introducing the questionnaires and were encouraged to answer questions clearly and completely. Moreover, many patients from both groups were under PPI therapy. This could impact the symptoms severity, QoL and the esophagitis grade. Since our study represents real-life clinical practice in which most GERD patients already treated with PPIs when they consult with a gastroenterologist, we were unable to only include patient’s off-PPIs. We have not applied the questionnaires at the baseline endoscopy; therefore, we are unable to perform comparison to baseline symptoms and QoL. Moreover, we used a disease specific questionnaire to assess QoL, which might not entirely capture all aspects of QoL, therefore future validation studies of these questionnaires would benefit from adding a general QoL questionnaire such as SF-36. Finally, our study had a modest sample size of 11 patients who fill in the Hebrew version of the questionnaires. This encourages future validation studies with a larger sample size in the Jewish population.

The strengths of our study include a well-defined of endoscopically classified EE patients, unique ethnic groups, and the validation of the QoL and RDQ tools, which will enable more in-depth research of GERD in these and other populations.

In conclusion, the RDQ and GERD QoL Arabic and Hebrew versions are valid and reliable for implementation in clinical practice in Israeli population. The clinical utility of these questionnaires is mainly for clinical follow up. GERD symptoms severity was more profound among patients with more severe esophagitis. We found no significant association between EE grade and QoL, which reflects negative results in this component of the study, possibly due to the improvement in esophagitis endoscopic findings a few years after the baseline assessment among patients with C/D grade.

### Supplementary Information

Supplementary Information.

## Data Availability

The datasets used and/or analysed during the current study are available from the corresponding author upon reasonable request.

## Abbreviations

BMI: Body mass index
CI: Confidence interval
EE: Erosive esophagitis
GERD: Gastro-esophageal reflux disease
NERD: Non-erosive reflux esophagitis
OR: Odds ratio
PPI: Proton pump inhibitors
QoL: Quality of life
RDQ: Reflux disease questionnaire
SD: Standard deviation
