# Supplementary material for: Quality of life and severity of symptoms among patients with various degrees of reflux esophagitis: a prospective study
Source: Sci Rep. 2023 Aug 26;13:13970. doi: 10.1038/s41598-023-41332-w (PMC10460377; doi:10.1038/s41598-023-41332-w)
Supplement: Supplementary file 1 — Supplementary Information. [file 41598_2023_41332_MOESM1_ESM.docx]

**Supplementary Table 1: Comparison of severity scores of symptoms using the RDQ**

**between patients with EE grades C/D and those with grade A/B**

|  | **EE grades A/B (N=99)**  **Median (IQR)** | **EE grades**  **C/D (N=50)**  **Median (IQR)** | **P value *** |
| --- | --- | --- | --- |
| **Overall symptoms severity scale** | 0.3 (1.1) | 0.6 (1.3) | 0.1 |
| **Regurgitation scale** | 0.0 (1.0) | 0.6 (1.5) | 0.04 |
| **Heartburn scale** | 0.0 (1.3) | 0.3 (1.5) | 0.2 |
| **Dyspepsia scale** | 0.0 (1.2) | 0.0 (1.3) | 0.3 |

EE: erosive esophagitis. * P value by the Mann-Whitney U test
